# Conjunctival Swab Findings in 484 COVID-19 Patients in Four Hospital Centers in Slovakia

**DOI:** 10.3390/vision6030046

**Published:** 2022-07-22

**Authors:** Alena Furdova, Pavol Vesely, Michal Trnka, Elena Novakova, Michal Stubna, Robert Furda, Lubica Branikova, Zuzana Pridavkova

**Affiliations:** 1 Department of Ophthalmology, Faculty of Medicine, Comenius University, 826 01 Bratislava, Slovakia; palo.vesely@veselyok.com; 2 VESELY Eye Clinic, 826 00 Bratislava, Slovakia; 3 Department of Medical Physics, Biophysics, Informatics and Telemedicine, Faculty of Medicine, Comenius University, 821 01 Bratislava, Slovakia; michal.trnka@fmed.uniba.sk; 4 Department of Microbiology and Immunology, Jessenius Faculty of Medicine, Comenius University, 036 01 Martin, Slovakia; elena.novakova@uniba.sk; 5 Department of Ophthalmology, Faculty Hospital, 010 01 Zilina, Slovakia; michael.st.md@gmail.com; 6 Department of Information Systems, Faculty of Management, Comenius University, 820 05 Bratislava, Slovakia; furda7@uniba.sk; 7 Department of Ophthalmology, Faculty Hospital, 940 62 Nove Zamky, Slovakia; lubicabranikova@yahoo.com; 8 Uvea Mediklinik, 036 00 Martin, Slovakia; zuzana.pridavkova@gmail.com

**Keywords:** COVID-19, polymerase chain reaction, conjunctival sac, lacrimal apparatus, analysis

## Abstract

Since 2020, the COVID-19 (Coronavirus Disease 2019) has quickly become a worldwide health problem. Ophthalmologists must deal with symptoms as well. For the positive detection in the conjunctival sac swab in COVID-19 patients hospitalized in Slovakia during March 2021 in four hospital centers, we used a test based on a polymerase chain reaction (PCR). In a group of 484 patients, 264 males (55%) and 220 females (45%) with clinical symptoms were identified with COVID-19 as a clinical diagnosis. The PCR test swab results from the conjunctival sac taken on the same day were positive in 58 patients (12%), 31 males (with a mean age of 74.6 ± 13.59 years) and 27 females (with a mean age of 70.63 ± 14.17 years); negative in 417 patients (86%); and 9 patients (2%) had an unclear result. The cycle threshold values comparing the nasopharynx and conjunctiva were also different in the group of all patients divided by age and gender. In COVID-19 patients the severe acute respiratory syndrome coronavirus-2 (SARS-CoV-2) was detectable using PCR test in the nasopharynx but also in the conjunctival sac swab, where the positivity rate was only 12%.

## 1. Introduction

The novel COVID-19 (Coronavirus Disease 2019) appeared in 2020 and is caused by the severe acute respiratory syndrome coronavirus-2 (SARS-CoV-2). Since 2020, this disease has quickly become a worldwide health problem, and ophthalmologists also have to deal with its symptoms [1,2].

The basic problem is represented by respiratory symptoms and myalgias, but in addition, the “red eye problem” and/or conjunctivitis was recognized as a supplementary clinical manifestation that was associated with SARS-CoV-2 infection [3,4].

The conjunctival epithelium or retina has the necessary proteins, for example, angiotensin-converting enzyme 2 (ACE2), transmembrane serine protease 2 (TMPRSS2), CD147, and Cathepsin L (CTSL) to potentially be infected with SARS-CoV-2. Additionally, regarding direct conjunctival ocular infection, the virus carried by tears through the nasolacrimal pathways to the nose represents a means of ocular inoculation. The evidence is that SARS-CoV-2 may either directly infect cells on the conjunctival or ocular surface [5].

In some studies, published before 2020, the presence of SARS-CoV-2 ribonucleic acid (RNA) in tears and the conjunctival sac was described in COVID-19 patients [6,7,8,9].

It is well known that the primary paths of SARS-CoV-2 infection transmission are confirmed through respiratory droplets. The first published data included clinical symptoms such as “red eye” and conjunctivitis that were observed in COVID-19 patients [9,10].

This study aims to evaluate the positivity of conjunctival sac swabs using a polymerase chain reaction (PCR) test in COVID-19 patients investigated by us and to extend the preciseness of the results that is described in our previous study [2].

## 2. Materials and Methods

The group of 484 investigated patients with a confirmed clinical diagnosis of COVID-19, the Alpha variant, were from four hospital centers in Slovakia (Bratislava, Nitra, Nove Zamky, and Zilina). All patients were hospitalized, and in all, a positive nasopharyngeal PCR test was confirmed. The clinical symptoms, including the ocular symptoms, have been documented in the patient records.

Within 3 days of admission to the hospital, patients underwent the conjunctival sac swab PCR testing for COVID-19. The used conjunctival sac swab used was a polyester (Dacron) swab with the NADAL^®^ COVID-19 IgG/IgM (the manufacturer: Nal von Minden GmbH, Moers, Germany) that was analyzed by PCR test. All conjunctival sac swabs, gathered by ophthalmologists from February to March 2021, were stored for 24 h, and the consequent analysis was performed in a dedicated laboratory of the Department of Microbiology and Immunology, Jessenius Faculty of Medicine, Comenius University, in the town of Martin, Slovakia.

The isolation of RNA from all conjunctival sac swabs, containing the concentrated virus, was performed using the Quick-RNA™ Viral 96 kit (catalog number R1041 from the manufacturer Zymo Research, Irvine, CA, USA), but as recommended by the manufacturer, the following modification was performed: 200 μL of DNA/RNA Shield^TM^ (manufacturer: Zymo Research, Irvine, CA, USA) was added to 200 μL of the transport medium that contained the conjunctival sac swab. After the modification application, the conjunctival sac swabs were mixed with viral RNA buffer and shifted to the Zymo-Spin™ I-96 Plate (manufacturer: Zymo Research, Irvine, CA, USA) to perform the washing and centrifugation. Then, the viral RNA was shifted with 25 μL DNase/RNase-free water up to the elution plate to perform the PCR test.

For the PCR test, the Multiplexed rTest set (rTEST COVID19/FLU qPCR Kit from the manufacturer MultiplexDX™ International, Rockville, MD, USA) was used according to the manufacturer’s instructions. The PCR was performed on the qTOWER^3^ (the manufacturer: Analytic Jena GmbH, Jena, Germany) using the standard Thermal Cyclers. The viral load, which was related to the initial conjunctival sac swab, was estimated within the specification of the cycle threshold (C_T_) for SARS-CoV-2. The final results were analyzed with the real-time PCR control and evaluation software (qPCRsoft, manufacturer: Analytik Jena AG, Jena, Germany) by performing the constant threshold calculation to determine the C_T_ values.

### Statistical Analysis

For the comparisons of the means of the defined groups, the paired t-test for normally distributed continuous variables was used. The significance level for all tests was carried out with the α = 0.001, where the two-tailed p-value, lower than 0.001, was considered statistically significant. For the data analysis, the statistical software IBM SPSS, version 27, was used (the vendor: IBM SPSS Inc., Armonk, NY, USA).

## 3. Results

In the group of 484 patients, 264 males (55%) and 220 females (45%) with the diagnosis of COVID-19 the mean age value was 66.69 ± 13.59 years, in the range from 25 to 96. For the 264 male patients, the median age value was 66 years and the mean age value was 65.59 ± 13.43 years. For the 220 female patients, the median age value was 69 years and the mean age value was 68.01 ± 13.71 years. Of the 484 hospitalized patients in inpatient care, 394 patients (81.2%) were treated with non-mechanically ventilated oxygen and 11 patients (2.3%) were treated with mechanically ventilated oxygen daily for 24 h. The PCR test results from the conjunctival sac swab, taken on the same day, were positive in 58 patients (12%), where 31 males had a mean age value of 74.6 ± 13.59 years and 27 females had a mean age value of 70.63 ±1 4.17 years; were negative in 417 patients (86%); and 9 patients (2%) had an unclear test result. Of 58 positives, 48 patients were treated with non-mechanically ventilated oxygen and 2 patients were treated with mechanically ventilated oxygen. Symptoms, such as “red eye” and the presence of conjunctivitis, were taken from patient records in 59 patients (Table 1).

**Figure 1:**
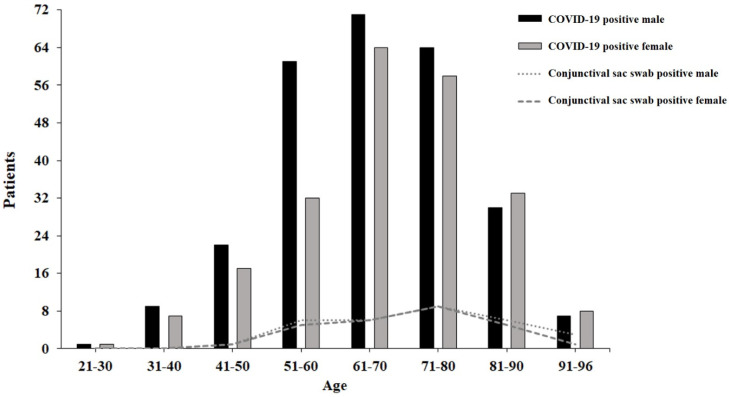
Patients in the group with COVID-19-positive PCR test and patients with positive result from the conjunctival sac divided by age and gender.

In the graph (Figure 1) we can see the group of all patients with a COVID-19-positive PCR test and patients with a positive result from the conjunctival sac divided by age and gender.

The male group had 264 patients, where 1 (0.2%) was in the age range 21–30 years, 9 (1.9%) were in the age range 31–40 years, 22 (4.6%) were in the age range 41–50 years, 60 (12.4%) were in the age range 51–60 years, 71 (14.7%) were in the age range 61–70 years, 64 (13.2%) were in the age range 71–80 years, 30 (6.2%) were in the age range 81–90 years, and 7 (1.4%) were in the age range 91 and more years. The female group had 220 patients, where 1 (0.2%) was in the age range 21–30 years, 7 (1.4%) were in the age range 31–40 years, 17 (3.5%) were in the age range 41–50 years, 32 (6.6%) were in the age range 51–60 years, 64 (13.2%) were in the age range 61–70 years, 58 (12%) were in the age range 71–80 years, 33 (6.8%) in the age range 81–90 years, and 8 (1.6%) were in the age range 91 or more years (Table 2).

In the group of all patients with a positive result from the conjunctival sac, the C_T_ values were significantly different when comparing the nasopharynx and conjunctiva, with the mean values of 22.71 ± 5.01 and 30.98 ± 4.19 with *p* < 0.001 (Table 3).

In the group of all patients divided by age and gender, the C_T_ values were different when comparing nasopharynx and conjunctiva (Table 4).

The sampling, conducted at four different hospital centers (towns) throughout Slovakia, documented the different numbers of patients with confirmed SARS-CoV-2 at each sampling site. Most swabs from the conjunctival sac were performed in the town Bratislava (149) with the most samples with a positive result (25). The next in the number of swabs was the town Nove Zamky (119 with 15 positives), the third was the town Zilina (117 with 9 positives), and the last one was the town Nitra (99 with 9 positives) (Table 5).

## 4. Discussion

According to recent studies, coronaviruses have rarely been associated with conjunctivitis or adnexal ocular surface infection [11,12]. Up to 2005, only a few reports of conjunctivitis were associated with HCoV-NL63 (the human coronavirus NL63), but no studies have been reported for the SARS-CoV-2 virus [13,14]. The mechanism of how these types of viruses can spread through the eye surface is still unknown. In a study in a group of 36 patients with the SARS disease, the coronavirus was detected in the conjunctiva in three cases [15], but in other studies, the swab was negative [16,17]. The question of triggering the mechanisms and details of the identification of the virus in ocular adnexal tissues and tears is still unknown [18,19]. Two studies showed a low rate of SARS-CoV-2 virus detection in conjunctiva—only about 4% [7,20]. Additionally, in a large meta-analysis, including a group of over one thousand patients, the frequency of conjunctivitis associated with SARS-CoV-2 infection was about 1% [21].

However, recent studies showed an expression of established SARS-CoV-2 and SARS-CoV-2 receptors—the ACE2 and TMPRSS2 (transmembrane protease serine subtype 2) in the ocular surface tissues (conjunctiva, cornea, and limbus) [22,23,24,25]. The results of these studies indicate that adnexal ocular surface cells could be susceptible to the virus and could serve as a point of entry for the virus. However, there is still an open question as to why, if the receptors for the virus are present in ocular surface tissues, the incidence of ocular surface infection is not higher, as it was also very low in our study.

One of the first published studies in 2020 [9] demonstrated that SARS-CoV-2 is detectable in the conjunctival swab confirmed with COVID-19. The results showed that the positivity rate in detecting SARS-CoV-2 in the conjunctival swabs was low, and out of the group of forty-five patients, only one patient (2.23%) was positive, with a C_T_ value of 33, using a real-time reverse transcription polymerase chain reaction for SARS-CoV-2. They did not describe any eye symptoms in this study. To compare with a higher preciseness, we extended our previous study [2]. So, in this paper, we extended the scope to evaluate 484 patients by including the stratification by age range (decades) and gender. The percentage of patients with positive results from the conjunctival sac increased to 11.98%. We found the eye symptoms (for example, itching, red eye, and others) in 59 patients (12.2%), which is an increase in percentage by more than 1% from our previous study. To complement the statistical results, we calculated the C_T_ values comparing the nasopharynx and conjunctiva stratified by age range and gender.

SARS-CoV-2 gains passage through angiotensin-converting enzyme 2 receptor that may be expressed in several tissues and may include the conjunctiva too [25]. More studies have reported the growing trend that, in some cases, COVID-19 pneumonia has been started alongside conjunctivitis after contact with infected persons. The discovery of viral RNA by RT-qPCR can be of value during the premature discovery of COVID-19 and when taking appropriate insulation measures. After that, defining whether SARS-CoV-2 is transmissible if it touches the conjunctival sac then it is considered to warrant significant investigation [26,27,28].

In some animal model studies, it has been reported that coronavirus transmission can occur via ocular inoculation, for example, ferrets may develop conjunctivitis after ocular inoculation with SARS-CoV-1 or pigs, mice, rats, or cats may develop a systemic coronavirus infection after transocular-transconjunctival inoculation [29,30,31,32,33,34].

Other sources documented that SARS-CoV-2 RNA has been recognized in the tears of infected COVID-19 patients, but many cases did not report conjunctivitis. Therefore, the benefit of gathering tears and ocular discharge for SARS-CoV-2 detection seems limited [35,36].

In the study by Gunduz [37], the simultaneous conjunctiva and oropharynx-nasopharynx swabs in patients who had presented to the outpatient department with the suspicion of SARS-CoV-2 syndromes were studied. An oropharynx–nasopharynx sample was obtained following bilateral conjunctiva swabs in 85 patients with no ocular symptoms or findings but with a contact history or more common symptoms, albeit with unknown SARS-CoV-2 status. Their results were evaluated according to the patient’s symptoms and the technique of how the swab was taken. The conjunctiva swab was positive in 29 (34.1%) cases and the oro–nasopharynx swab in 20 (23.5%) cases. In both methods, positive results were in 11 (14.1%) cases. The mean CT value was 30.15 ± 3.41 in symptomatic cases and 33.62 ± 1.76 in asymptomatic cases (*p* = 0.008). In cases that were positive using both methods, the mean CT value was 25.21 ± 4.94 using the oro–nasopharynx swab and 30.29 ± 5.05 using the conjunctiva swab. The researchers found higher SARS-CoV-2 detection rates with the conjunctiva swab than with the oro–nasopharynx swab in patients with unknown SARS-CoV-2 status. Due to their results, the conjunctival viral load appeared higher in symptomatic cases than in asymptomatic cases. They believe a conjunctiva swab could be an alternative method of detecting SARS-CoV-2 at the point of the initial presentation to the outpatient department.

Throughout the COVID-19 pandemic, more and more studies have reported and highlighted several signs of this infection in the ocular area. As the result of the discovery, a targeted meta-analysis documented the overall preponderance of ocular symptoms at occurrence 11% [38]. In this study, the authors documented the most frequently appearing ocular symptoms in COVID-19 patients, such as ocular pains, follicular conjunctivitis, and redness. As seen in other studies, SARS-CoV-2 can be verified using a swab in the conjunctival secretion with consequent analysis by RT-PCR. We can state the same results in our study.

Only a few authors have proposed a protection implementation for the eye in order to avoid the expected COVID-19 transmission. The suggestions for the protection implementation include various techniques that can successfully foresee the transmission possibilities of COVID-19 from patient to ophthalmologist or optometrist. They should be formed from ocular systems and should cover various activities, for example, cataract surgery, intraocular pressure measuring procedures by non-contact tonometry, and others [39,40,41,42,43,44].

Regarding the discussed topics, we also include data from two studies dealing with meta-analysis that uncovered a number of different eye problems, such as visual acuity decline, eye redness, follicular conjunctivitis, and others [20,44].

In different studies on COVID-19 patients, various eye symptoms, for example, dry eye, “red eye”, and foreign body sensation, have often been present. Some studies have been included more refined on detailing different indications, for example, from healthcare workers. In practice, eye symptoms can be overseen because of the more extreme clinical indicators. Therefore, eye symptoms have rarely been noticed. Some studies have elevated the concerns that COVID-19 may have ocular manifestations in the form of follicular conjunctivitis as the first single symptom of this disease. Summary information from the three meta-examinations revealed that visual side-effects may be a major concern in approximately 2.2% of patients [44,45,46,47,48].

Generally, eye symptoms seem to be correlated with the high viral titer of conjunctival sac swab results. In our findings, we did not recognize any correlation between the titter of PCR and ocular symptoms because in conjunctival sac swab-positive patients, we did not always find eye symptoms. Similar percentages of conjunctival sac swab-positive and -negative patients showed eye symptoms. The authors of one systematic review compared more than two hundred ophthalmology-focused scientific articles dealing with the COVID-19 pandemic that were published throughout the last few months and reported the presence of conjunctivitis by including other eye manifestations [49].

In our study, conjunctival sac swabs with PCR testing were performed on 484 patients with COVID-19 (mean age of 66.7 years). PCR positivity increased with age in both sexes, peaking in patients in their 70s and decreasing in patients in their 80s and 90s. There is no causal relationship between severity and PCR positivity, but the overall positivity rate was 12%, which is higher than in previous reports. We documented that in patients with COVID-19 there is a pathway for SARS-CoV-2 transmission through ocular exposure, but we cannot prove the original direction of infection between the ocular surface tissue and the body of the patients. Our study was limited in methodology in two ways. In the four different hospital centers for COVID-19, the swabs were performed by more than two ophthalmologists, which can result in different approaches to taking swabs from the conjunctival sac, and also because full-body anti-infection clothing partially interfered with this activity. The clinical eye symptoms of conjunctivitis were not directly investigated but were only taken from the patients’ records and patients’ history.

## 5. Conclusions

By examining the results of our multi-center study, we contributed to this speedily emerging topic. The execution of our study contributes to the confirmation that in COVID-19 patients, SARS-CoV-2 is also detectable with the conjunctival sac swab by PCR test in patients who have had a PCR-positive nasopharyngeal swab, but the positivity rate is only about 12%. Currently, we think that our results of the PCR positivity results from conjunctival sacs are not applicable to clinical practice, but in the future, we believe the results will be used for clinical and other purposes.

## Figures and Tables

**Table 1:** The overview of all patients with COVID-19-positive PCR test and the patients with a positive result from the conjunctival sac.

Gender	Number	Gestational Age ^a^ (Years)	Non-Mechanically Ventilated Patients ^b^	Mechanically Ventilated Patients ^b^	Positive Result from the Conjunctival Sac	Eye Symptoms ^b^
Male	264	65.59 ± 13.43	215 (26)	5 (0)	31	36 (4)
Female	220	68.01 ± 13.71	179 (22)	6 (2)	27	23 (4)
Overall	484	66.69 ± 13.59	394 (48)	11 (2)	58	59 (8)

^a^ Mean and Standard Error of Mean (SEM) have been calculated and are shown as Mean ± SEM ^b^ Patients (Patients with positive result from the conjunctival sac).

**Table 2:** The overview of all patients with COVID-19-positive PCR test and the patients with a positive result from the conjunctival sac divided by age and gender.

Age Range (Years)	Male ^a^	Female ^a^	Non-Mechanically Ventilated Patients ^b^	Mechanically Ventilated Patients ^b^	Eye Symptoms ^b^
21–30	1 (0)	1 (0)	1 (1)	0 (0)	0 (0)
31–40	9 (0)	7 (0)	8 (5)	0 (0)	0 (0)
41–50	22 (1)	17 (1)	16 (13)	0 (1)	8 (1)
51–60	60 (6)	32 (5)	46 (25)	2 (3)	5 (8)
61–70	71 (6)	64 (6)	49 (55)	3 (1)	9 (9)
71–80	64 (9)	58 (9)	59 (45)	0 (1)	9 (3)
81–90	30 (6)	33 (5)	29 (29)	0 (0)	5 (1)
>91	7 (3)	8 (1)	7 (6)	0 (0)	0 (1)

^a^ Patients (Patients with a positive result from the conjunctival sac) ^b^ Male (Female).

**Table 3:** The cycle threshold (C_T_) values from the group of all patients with positive result from the conjunctival sac.

Gender	Number	C_T_ Value in Nasopharynx ^a^	C_T_ Value in Conjunctiva ^a^	*p*
Male	27	22.98 ± 5.14	29.53 ± 4.11	<0.001
Female	31	22.47 ± 4.98	32.32 ± 3.87	<0.001
Overall	58	22.71 ± 5.01	30.98 ± 4.19	<0.001

^a^ Mean and standard error of mean (SEM) were calculated and are shown as mean ± SEM.

**Table 4:** The cycle threshold (C_T_) values from the group of all patients with a positive result from the conjunctival sac divided by age and gender.

Age Range (Years)	Male			Female		
	Number	C_T_ Value in Nasopharynx ^a^	C_T_ Value in Conjunctiva ^a^	Number	C_T_ Value in Nasopharynx ^a^	C_T_ Value in Conjunctiva ^a^
40–49	1	29.80 ± 0.00	33.54 ± 0.00	1	18.50 ± 0.00	32.02 ± 0.00
50–59	6	20.87 ± 5.08	31.13 ± 4.12	5	21.78 ± 5.41	35.35 ± 2.17
60–69	6	21.47 ± 6.60	31.11 ± 4.97	6	26.39 ± 5.01	33.28 ± 5.08
70–79	9	24.06 ± 6.33	29.81 ± 3.92	9	21.96 ± 4.62	33.05 ± 4.22
80–89	6	23.60 ± 3.22	29.09 ± 5.82	5	22.49 ± 4.12	31.41 ± 1.96
>90	3	21.51 ± 4.78	29.99 ± 3.62	1	14.00 ± 0.00	22.56 ± 0.00

^a^ Mean and standard error of mean (SEM) were calculated and are shown as mean ± SEM.

**Table 5:** Monitored parameters from four hospital centers (towns) and overall, with statistical results.

	Bratislava		Zilina		Nitra		Nove Zamky		Overall	
	Number	% ^a^	Number	% ^a^	Number	% ^a^	Number	%	Number	% ^a^
Male	76	51.01	70	59.83	58	58.59	60	50.42	264	54.55
Female	73	48.99	47	40.17	41	41.41	59	49.58	220	45.45
Overall	149	100	117	100	99	100	119	100	484	100
Positive ^d^ overall	25	16.78	9	7.69	9	9.09	15	12.61	58	11.98
Positive ^d^ male	13	8.72	3	2.56	7	7.07	8	6.72	31	6.40
Positive ^d^ female	12	8.05	6	5.13	2	2.02	7	5.88	27	5.58
Negative ^d^ overall	115	77.18	108	92.31	90	90.91	104	87.39	417	86.16
Negative ^d^ male	58	38.93	67	57.26	51	51.52	52	43.70	228	47.11
Negative ^d^ female	57	38.26	41	35.04	39	39.39	52	43.70	189	39.05
Unclear overall	9	6.04	-		-		-		9	1.86
Unclear male	5	3.36	-		-		-		5	1.03
Unclear female	4	2.68	-		-		-		4	0.83
Eye symptoms male	1	0.67	27	23.08	5	5.05	3	2.52	36	7.42
Eye symptoms female	1	0.67	14	11.97	6	6.06	2	1.68	23	4.74
Eye symptoms overall	2	1.33	41	35.04	11	11.11	5	4.20	59	12.16
	Number ^b^		Number ^b^		Number ^b^		Number ^b^		Number ^b^	
Mean duration ^c^ of interval in all patients	6.13 ± 4.86		7.20 ± 3.18		13.53 ± 7.13		10.79 ± 6.26		9.01 ± 6.14	
Mean duration ^c^ of interval in positive ^d^ patients	5.88 ± 4.68		5.89 ± 2.57		13.11 ± 7.72		9.50 ± 5.52		7.85 ± 5.78	
Mean duration ^c^ of interval in negative ^d^ patients	6.27 ± 4.92		7.31 ± 3.21		13.57 ± 7.12		10.98 ± 6.36		9.26 ± 6.18	
Average age	70 ± 13.09		62.41 ± 13.60		65.59 ± 12.67		67.71 ± 13.86		66.67 ± 13.59	
The average age of the positive ^d^ patients	73.76 ± 14.11		69.00 ± 13.35		65.33 ± 10.97		71.33 ± 9.51		71.09 ± 12.53	
The average age of the negative ^d^ patients	68.71 ± 12.66		61.86 ± 13.53		65.62 ± 12.88		67.19 ± 14.34		65.85 ± 13.58	

^a^ Percentages from each point separately. ^b^ Mean and standard error of mean (SEM) were calculated and are shown as mean ± SEM. ^c^ Days between the PCR tests from the nasopharynx and conjunctival sac. ^d^ Result from the conjunctival sac.

## Data Availability

The dataset that was used and/or analyzed during this study will be available from the corresponding author upon reasonable request.
